# Revisiting the taxonomy of Korean *Ischnochiton* species (Polyplacophora, Ischnochitonidae) based on a combined analysis of morphological and molecular data

**DOI:** 10.3897/BDJ.12.e134521

**Published:** 2024-10-23

**Authors:** Jina Park, Yukyung Kim, Eggy Triana Putri, Joong-Ki Park

**Affiliations:** 1 Division of EcoScience, Ewha Womans University, Seoul, Republic of Korea Division of EcoScience, Ewha Womans University Seoul Republic of Korea

**Keywords:** *Ischnochiton*, Ischnochitonidae, microstructures, tegmentum sculpture, girdle scales, radula, SEM, mtDNA *cox1*, Korea

## Abstract

**Background:**

Chiton species belonging to the genus *Ischnochiton* J. E. Gray, 1847 are commonly found in intertidal rocky shores worldwide, with the exception of the northern Atlantic and Arctic oceans. *Ischnochiton* species are characterised by imbricate girdle scales that are uniform in size, rounded, sculpted with striae or occasionally smooth. However, their species-level taxonomy is complicated due to the high variation in their shell microstructures. Despite more than a hundred species reported worldwide, taxonomic studies of this group remain relatively unexplored in Korean waters, with only a few species recorded to date.

**New information:**

In this study, we compared the microstructural characteristics of tegmentum sculpture, girdle scales and radula amongst four Korean *Ischnochiton* species using high-resolution microscopic images and a scanning electron microscope (SEM). Along with mtDNA *cox1* sequence comparison, a comprehensive analysis of their morphology revealed that *I.hayamii* Owada, 2018 was identified for the first time in Korean waters. This species is morphologically distinguished by its small body size of adults, smooth lateral areas on valves and small perinotum scales sculptured with weak longitudinal ribs. Phylogenetic analysis of the mtDNA *cox1* sequence provides distinct resolution at the species level, but interrelationships amongst *Ischnochiton* species remain unresolved. Results from the morphological and molecular analyses presented in this study offer valuable taxonomic information for accurate species identification amongst closely-related *Ischnochiton* species.

## Introduction

Chiton species belonging to the genus *Ischnochiton* J. E. Gray, 1847 are commonly found on the undersurface of boulders in intertidal rocky shores worldwide, with the exception of the northern Atlantic and Arctic oceans (Kaas and Van Belle 1990). This genus is characterised by the presence of imbricate girdle scales, which are typically equal in size, rounded, sculpted with striae, but occasionally smooth (Kaas and Van Belle 1990). It comprises approximately 110 species worldwide (MolluscaBase 2023), including nine species found in the north-western Pacific (e.g. China, Japan, Russian Far East, Taiwan and Korea). This group has been relatively well-studied in Japan (Kaas and Van Belle 1990, Owada 2016, Owada 2018), but taxonomic diversity and their fauna remain relatively unexplored in Korean waters, with only a few species reported to date (National list of species of Korea 2022): *I.boninensis* Bergenhayn, 1933, *I.comptus* (Gould, 1859) and *I.hakodadensis* P. P. Carpenter, 1893. Moreover, like many other chiton species, previous morphological descriptions were incomplete and extensive variations in the microscopic structures of tegmentum sculptures, girdle scales and radula characters complicate accurate species identification amongst closely-related species. Due to these taxonomic challenges, a combination of morphological examination with molecular sequence analysis is considered highly effective for their species-level taxonomy (Owada 2018).

In this study, we examined both morphological and molecular sequence data of four Korean *Ischnochiton* species. For this, we performed a comprehensive analysis of morphological characters (i.e. microstructures of tegmentum sculpture, girdle scales and radula) using high-resolution microscopic images and a scanning electron microscope (SEM). In addition, we conducted a phylogenetic analysis of the mtDNA *cox1* sequences and compared them with other *Ischnochiton* species to infer their phylogenetic relationships.

## Materials and methods

**Figure 1.**
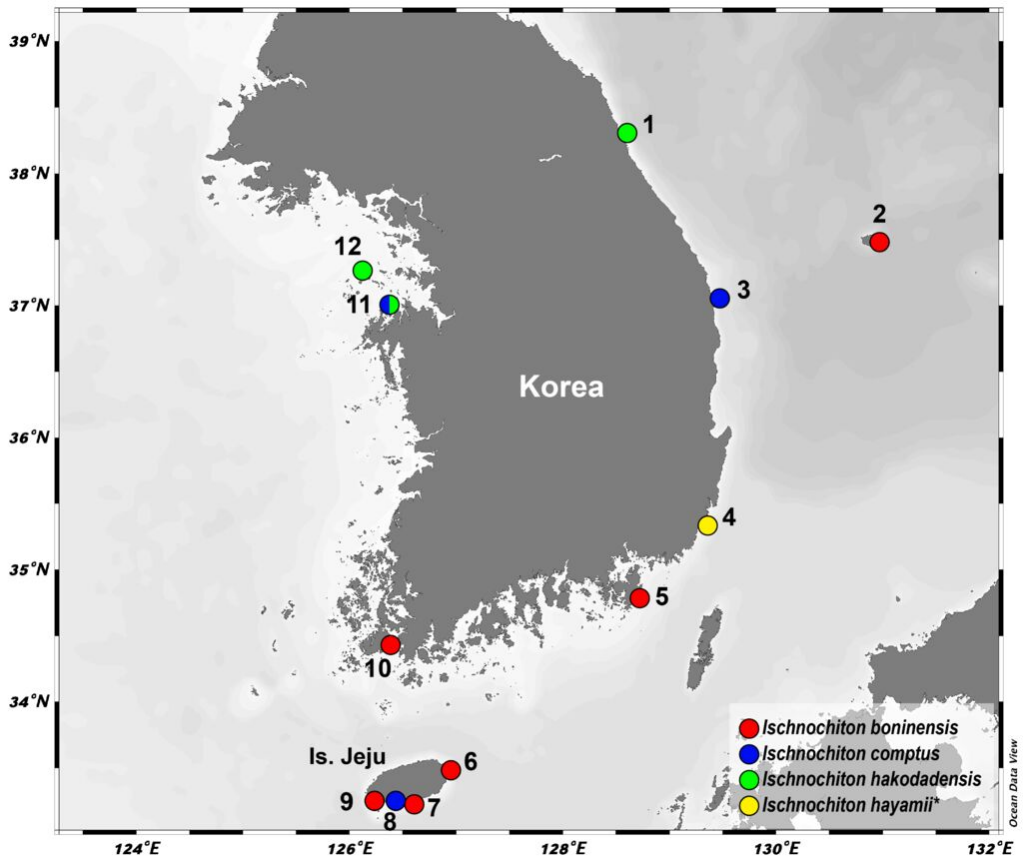
The map of sampling localities for Korean *Ischnochiton* species in this study. **1** Ayajin-ri, Toseong-myeon, Goseong-gun, Gangwon-do; **2** Dodong-ri, Ulleung-gun, Gyeongsangbuk-do; **3** Jukbyeon-ri, Jukbyeon-myeon, Uljin-gun, Gyeongsangbuk-do; **4** Sinam-ri, Seosaeng-myeon, Ulju-gun, Ulsan-si; **5** Gujora-ri, Irun-myeon, Geoje-si, Gyeongsangnam-do; **6** Jongdal-ri, Gujwa-eup, Jeju-si, Jeju-do; **7** Is. Mun, Seogwi-dong, Seogwipo-si, Jeju-do; **8** Andeok-myeon, Seogwipo-si, Jeju-do; **9** Daejeong-eup, Seogwipo-si, Jeju-do; **10** Geumgye-ri, Gogun-myeon, Jindo-gun, Jeollanam-do; **11** Dokgot-ri, Daesan-eup, Seosan-si, Chungcheongnam-do; **12** Buk-ri, Deokjeok-myeon, Ongjin-gun, Incheon-si. The species (*I.hayamii*) discovered for the first time in Korea is denoted by asterisks (*).

Specimens were collected from intertidal rocky shores and subtidal zones in Korean waters (Fig. 1) and preserved in 95% ethyl alcohol. For species identification, morphological characters of valves and girdle scales were examined using a stereoscopic microscope (Leica M205C; Wetzlar, Germany). Microstructures of tegmentum sculpture on the valves, girdle scales (perinotum and hyponotum) and radula were examined using a SEM. For SEM preparation, the valves, girdle and radula were dissected individually and incubated at 50℃ in a 10% potassium hydroxide (KOH) solution for approximately 5–10 min, then washed with distilled water. After washing, they were cleaned to remove any residual tissues using an ultrasonic cleaner (Shinhan 200H3L; Shinhan-Sonic, Korea), coated with platinum ions after drying and observed using a SEM (Ultra Plus; Zeiss, Germany). The abbreviations and morphological measures used in this study follow Saito 2004 and Schwabe 2010: ama, antemucronal area; ap, apophyses; ca, central area; c, central tooth; cl, centro-lateral tooth; e, eave; h, head of major lateral tooth; hs, hyponotum scale; im, inner marginal tooth; ip, insertion plate; isl, inner small lateral tooth; ja, jugal area; jl, jugal lamina; js, jugal sinus; la, lateral area; lmsp, large marginal spicule; m, mucro; mlt, major lateral tooth; mm, middle marginal tooth; mmsp, middle marginal spicule; mu, major uncinus tooth; om, outer marginal tooth; osl, outer small lateral tooth; pa, pleural area; pma, postmucronal area; pms, postmucronal slope; pp, petaloid process; pps, peripheral perinotum scale; ps, perinotum scale; sl, slit; slr, slit ray; smsp, small marginal spicule; t, tooth. The specimens examined for morphology and molecular analysis in this study were deposited in the National Institute of Biological Resources (NIBR voucher specimen nos. NIBRIV0000863005–NIBRIV0000863007, NIBRIV0000863018, NIBRIV0000863020–NIBRIV0000863022, NIBRIV0000863027–NIBRIV0000863030, NIBRIV0000863046–NIBRIV0000863048, NIBRIV0000863058, NIBRIV0000863095–NIBRIV0000863099 and NIBRIV0000863103) in Incheon and the Animal Phylogenomics Laboratory at Ewha Womans University in Seoul, Korea.

For the mtDNA *cox1* barcoding of the specimens, total genomic DNA was extracted from the foot tissue using the E.Z.N.A. Mollusc DNA kit (Omega Bio-tek, Norcross, USA) following the manufacturer’s protocols. To amplify a partial sequence of the mtDNA *cox1* gene, a polymerase chain reaction (PCR) was conducted using TaKaRa Ex Taq (Takara Bio, Shiga, Japan) with the universal primer set (LCO1490/HCO2198) (Folmer et al. 1994). The PCR was prepared in a total volume of 50 μl, consisting of 33.75 μl of distilled water, 5 μl of 10× Ex Taq buffer, 4 μl of dNTP Mixture (2.5 mM each), 2 μl of each primer (LCO1490/HCO2198), 0.25 μl of TaKaRa Ex Taq and 3 μl of genomic DNA template. The PCR conditions were as follow; an initial denaturation at 95°C for 1 min, followed by 40 cycles of denaturation at 94°C for 30 s, annealing at 46°C for 30 s, extension at 72°C for 30 s and a final extension at 72°C for 10 min. The amplified PCR products were isolated on a 1% agarose gel and purified using a QIAquick gel extraction kit (Qiagen, Valencia, CA, USA). Sequencing of the partial mtDNA *cox1* gene was performed bidirectionally using an ABI PRISM 3700 DNA analyser (Applied Biosystems, Foster City, CA, USA). The sequencing reaction using universal primers (LCO1490 and HCO2198) yielded a 658 bp mtDNA *cox1* sequence. However, some sequences obtained from GenBank were shorter than those generated in this study. Consequently, a 557 bp homologous region was prepared for subsequent analysis after trimming both ends of our sequences using Geneious Prime v. 2023.2.1 (Biomaters, Auckland, New Zealand).

Molecular analyses were conducted, based on a total of 85 mtDNA *cox1* sequences (23 and 62 sequences from this study and GenBank, respectively) of seven *Ischnochiton* species from the north-western Pacific (NWP) available on GenBank (Owada 2016, Owada 2018). Multiple sequence alignment was performed using MAFFT v. 7.490 (Katoh and Standley 2013) with default parameters in Geneious Prime v. 2023.2.1 (Biomaters, Auckland, New Zealand). Genetic distances (*p*-distance) between and within species were calculated using MEGA X (Kumar et al. 2018). A molecular phylogenetic tree was reconstructed in RAxML v.8.2.12 (Stamatakis 2014) using the Maximum Likelihood (ML) method with 1,000 bootstrap replications. The partial mtDNA *cox1* sequences of Korean *Ischnochiton* species determined in this study were deposited in GenBank (Table 1; PQ119731–PQ119753).

## Taxon treatments

### Ischnochiton boninensis

Bergenhayn, 1933

717A10D7-2C9F-514F-B545-D3DADC0AC1E5

https://www.marinespecies.org/aphia.php?p=taxdetails&id=387099

**Figure 2.**
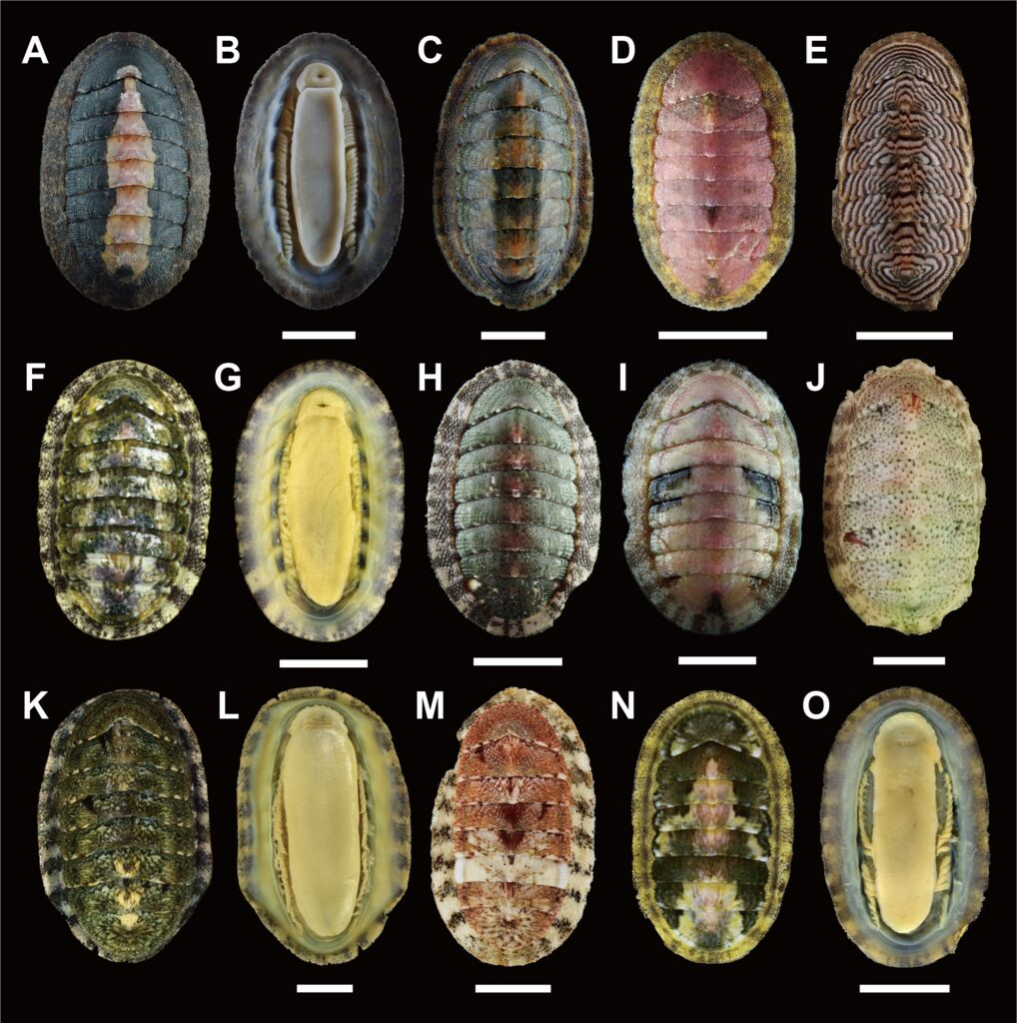
External view of Korean *Ischnochiton* species. **A** *I.boninensis* from Jeju, dorsal view; **B** *I.boninensis* from Jeju, ventral view; **C** *I.boninensis* from Ulleng (NIBRIV0000863007), dorsal view; **D** *I.boninensis* from Jeju, dorsal view; **E** *I.boninensis* (=*I.zebrinus*) from Jeju (NIBRIV0000863047), dorsal view; **F** *I.comptus* from Jeju (NIBRIV0000863098), dorsal view; **G** *I.comptus* from Jeju (NIBRIV0000863098), ventral view; **H** *I.comptus* from Jeju, dorsal view; **I** *I.comptus* from Jeju, dorsal view (NIBRIV0000863020); **J** *I.comptus* from Uljin, dorsal view (NIBRIV0000863048); **K** *I.hakodadensis* from Goseong (NIBRIV0000863028), dorsal view; **L** *I.hakodadensis* from Goseong (NIBRIV0000863028), ventral view; **M** *I.hakodadensis* from Goseong, dorsal view; **N** *I.hayamii* from Ulsan (NIBRIV0000863103), dorsal view; **O** *I.hayamii* from Ulsan (NIBRIV0000863103), ventral view. Scale bars: **A**–**O** = 5 mm. The NIBR voucher specimen numbers are provided in parentheses.

**Figure 3.**
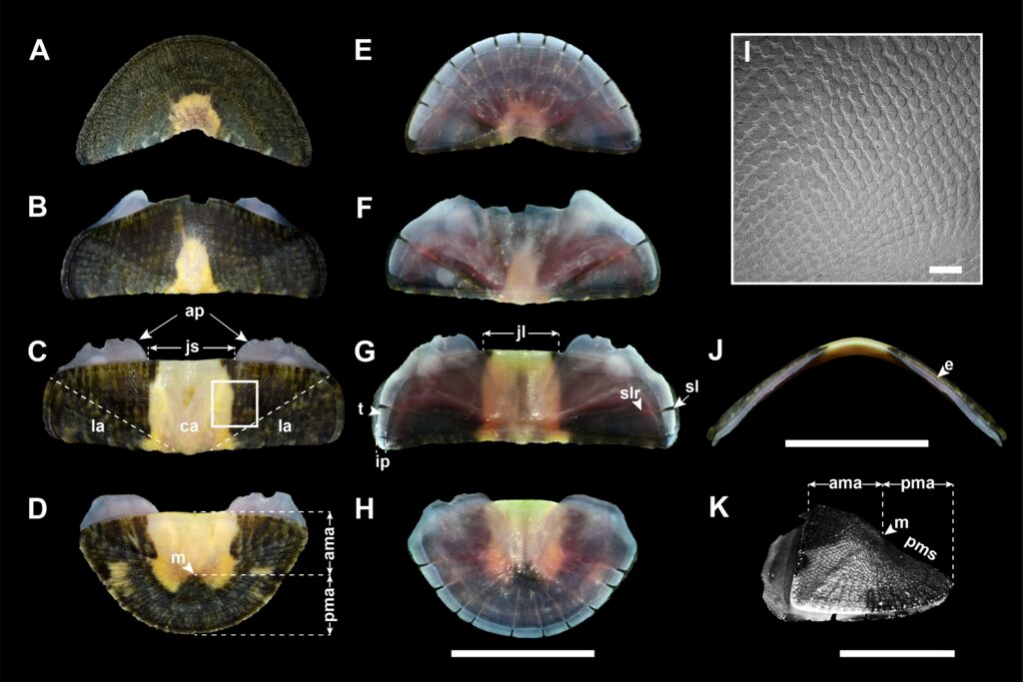
Valves of *I.boninensis* from Jeju (NIBR voucher specimen no. NIBRIV0000863097). **A** head valve, dorsal view; **B** 2^nd^ valve, dorsal view; **C** 4^th^ valve, dorsal view; **D** tail valve, dorsal view; **E** head valve, ventral view; **F** 2^nd^ valve, ventral view; **G** 4^th^ valve, ventral view; **H** tail valve, ventral view; **I** 4^th^ valve, detail of tegmentum surface of central area using scanning electron microscope; **J** 4^th^ valve, frontal view; **K** tail valve, lateral view. Abbreviations: ama, antemucronal area; ap, apophyses; ca, central area; e, eave; ip, insertion plate; jl, jugal lamina; js, jugal sinus; la, lateral area; m, mucro; pma, postmucronal area; pms, postmucronal slope; sl, slit; slr, slit ray; t, tooth. Scale bars: **A–H, J** = 5 mm, **I** = 200 μm, **K** = 2 mm.

**Figure 4.**
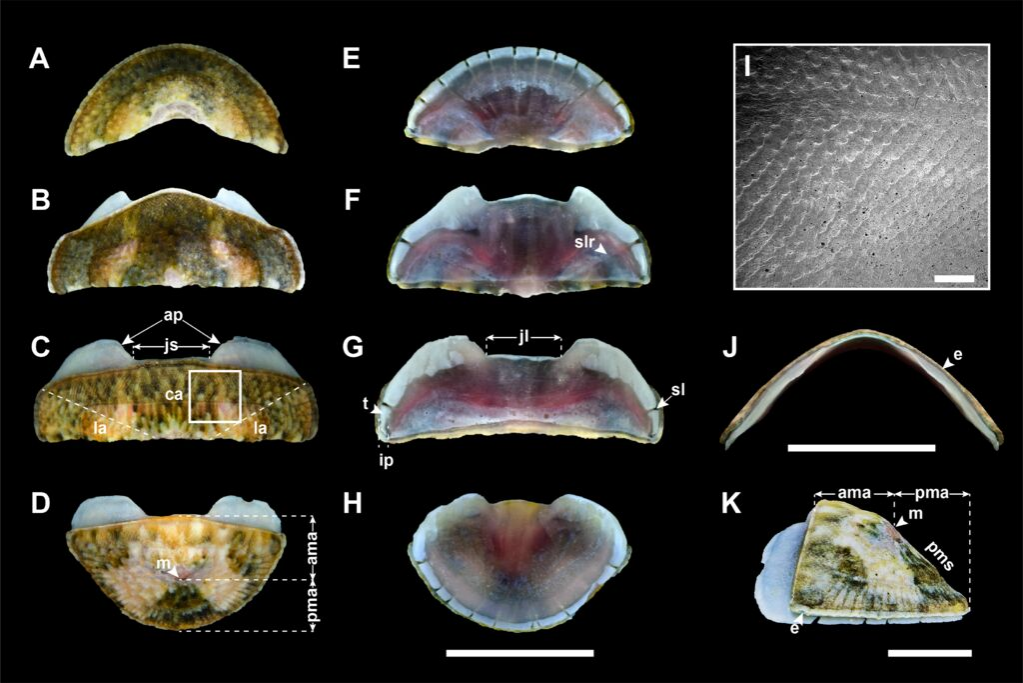
Valves of *I.comptus* from Jeju (NIBR voucher specimen no. NIBRIV0000863098). **A** head valve, dorsal view; **B** 2^nd^ valve, dorsal view; **C** 4^th^ valve, dorsal view; **D** tail valve, dorsal view; **E** head valve, ventral view; **F** 2^nd^ valve, ventral view; **G** 4^th^ valve, ventral view; **H** tail valve, ventral view; **I** 4^th^ valve, detail of tegmentum surface of central area using scanning electron microscope; **J** 4^th^ valve, frontal view; **K** tail valve, lateral view. Abbreviations: ama, antemucronal area; ap, apophyses; ca, central area; e, eave; ip, insertion plate; jl, jugal lamina; js, jugal sinus; la, lateral area; m, mucro; pma, postmucronal area; pms, postmucronal slope; sl, slit; slr, slit ray; t, tooth. Scale bars: **A–H, J** = 5 mm, **I** = 200 μm, **K** = 2 mm.

**Figure 5.**
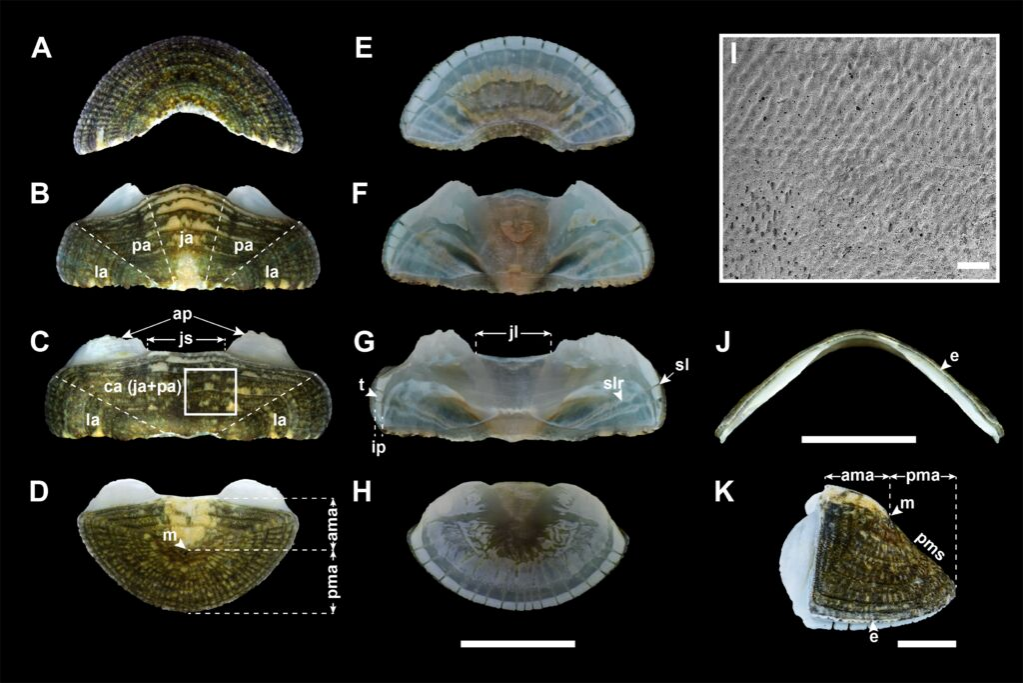
Valves of *I.hakodadensis* from Goseong (NIBR voucher specimen no. NIBRIV0000863046). **A** head valve, dorsal view; **B** 2^nd^ valve, dorsal view; **C** 4^th^ valve, dorsal view; **D** tail valve, dorsal view; **E** head valve, ventral view; **F** 2^nd^ valve, ventral view; **G** 4^th^ valve, ventral view; **H** tail valve, ventral view; **I** 4^th^ valve, detail of tegmentum surface of central area using scanning electron microscope; **J** 4^th^ valve, frontal view; **K** tail valve, lateral view. Abbreviations: ama, antemucronal area; ap, apophyses; ca, central area; e, eave; ip, insertion plate; ja, jugal area; jl, jugal lamina; js, jugal sinus; la, lateral area; m, mucro; pa, pleural area; pma, postmucronal area; pms, postmucronal slope; sl, slit; slr, slit ray; t, tooth. Scale bars: **A–H, J** = 5 mm, **I** = 200 μm, **K** = 2 mm.

**Figure 6.**
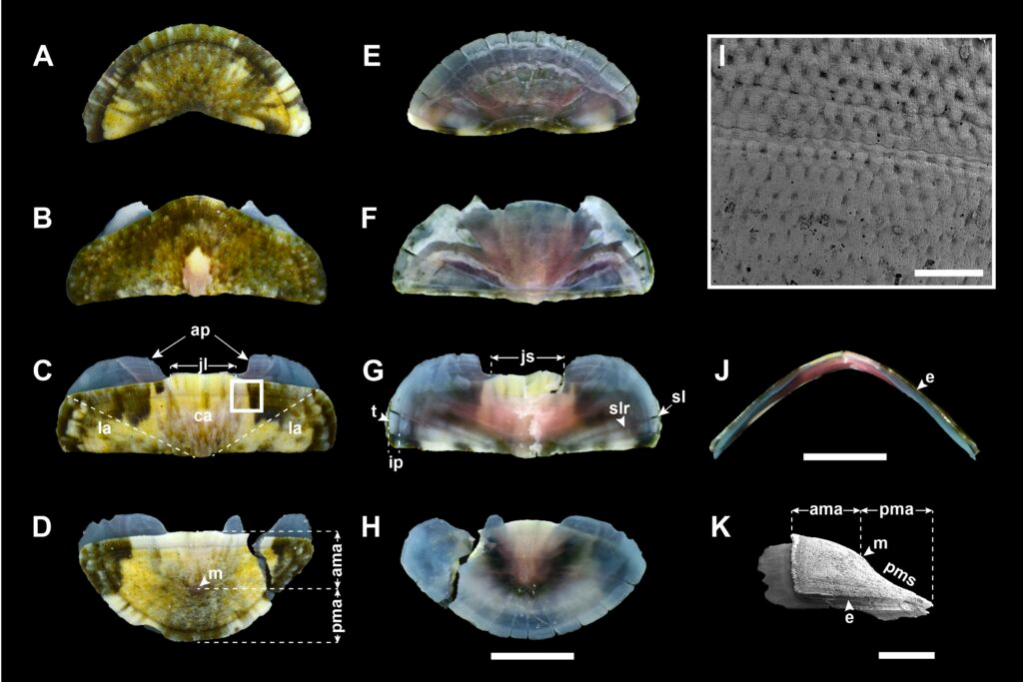
Valves of *I.hayamii* from Ulsan (NIBR voucher specimen no. NIBRIV0000863103). **A** head valve, dorsal view; **B** 2^nd^ valve, dorsal view; **C** 4^th^ valve, dorsal view; **D** tail valve, dorsal view; **E** head valve, ventral view; **F** 2^nd^ valve, ventral view; **G** 4^th^ valve, ventral view; **H** tail valve, ventral view; **I** 4^th^ valve, detail of tegmentum surface of central area using scanning electron microscope; **J** 4^th^ valve, frontal view; **K** tail valve, lateral view using scanning electron microscope. Abbreviations: ama, antemucronal area; ap, apophyses; ca, central area; e, eave; ip, insertion plate; jl, jugal lamina; js, jugal sinus; la, lateral area; m, mucro; pma, postmucronal area; pms, postmucronal slope; sl, slit; slr, slit ray; t, tooth. Scale bars: **A–H, J** = 2 mm, **I** = 200 μm, **K** = 1 mm.

Ischnochiton boninensis Bergenhayn (1933): 10–13, taf. 1, figs. 2, 3, taf. 2, figs. 24–29, 32, textfig. 3; Taki (1938): 369, 373, 375; Kang (1971): 52; Kaas and Van Belle (1980): 18; Kaas and Van Belle (1998): 33; Kira (1982): 183, pl. 67, fig. 2; Saito (1995): 105; Saito (2000): 12, 13, pl. 6, fig. 15; Saito (2017): 732, pl. 4, fig. 11; Slieker (2000): 98, 99, pl. 37, fig. 10; Owada (2016): 5, 6, figs. 4A–E, 5A–E; Sirenko and Zhang (2019): 6, fig. 2B.
Ischnochiton zebrinus Bergenhayn (1933): 13–15, taf. 1, fig. 4, taf. 2, figs. 30, 31, 33–39, textfig. 4; Taki (1938): 375; Taki (1964a): 342.
Ischnochiton (Ischnochiton) boninensis : Taki (1962): 43; *Higo (1973)*: 6; Yum (1988): 20, 21, pl. 1, fig. 4, pl. 8, pl. 23, figs. 1, 2, pl. 28, fig. 4; Kaas and Van Belle (1990): 184–186, fig. 83, map 39; Higo et al. (1999): 27; Lee and Min (2002): 94; Min et al. (2004): 70, 71, fig. 8.
Ischnochiton (Ischnochiton) comptus (Gould) forma isaoi : Taki (1964a): 348, text-figs. 2, 4, 6; Taki (1964b): 408; Higo (1973): 6; Higo and Goto (1993): 3.
?Ischnochiton (Ischnochiton) zebrinus: Taki (1962): 43; Taki (1964b): 408; Higo (1973): 6; Higo and Goto (1993): 3; Higo et al. (1999): 27.
Ischnochiton (Simplischnochiton) boninensis : Van Belle (1982): 470; Dell'Angelo et al. (1990): 36, 37.

#### Materials

- **Type status:** Other material. **Occurrence:** individualCount: 3; occurrenceID: B04F80DF-C510-566A-9E5A-CEEA8BBADF80; **Taxon:** scientificName: *Ischnochitonboninensis*; **Location:** country: Korea; locality: Jongdal-ri, Gujwa-eup, Jeju-si, Jeju-do; verbatimCoordinates: 33°29'16.84"N, 126°54'41.37"E; **Event:** eventDate: 26 Jan 2015
- **Type status:** Other material. **Occurrence:** individualCount: 5; occurrenceID: C8673915-9FF2-5CC2-AC00-144DD7F27CAD; **Taxon:** scientificName: *Ischnochitonboninensis*; **Location:** country: Korea; locality: Jongdal-ri, Gujwa-eup, Jeju-si, Jeju-do; verbatimCoordinates: 33°29'16.84"N, 126°54'41.37"E; **Event:** eventDate: 21 Dec 2015
- **Type status:** Other material. **Occurrence:** individualCount: 1; occurrenceID: 283FB9B8-108F-585F-B330-D280BD77DF60; **Taxon:** scientificName: *Ischnochitonboninensis*; **Location:** country: Korea; locality: Daejeong-eup, Seogwipo-si, Jeju-do; verbatimCoordinates: 33°14'27.09"N, 126°13'21.24"E; **Event:** eventDate: 02 Jul 2016
- **Type status:** Other material. **Occurrence:** individualCount: 2; occurrenceID: 7BB82550-EA20-5A66-931F-2117EB63AA04; **Taxon:** scientificName: *Ischnochitonboninensis*; **Location:** country: Korea; locality: Is. Mun, Seogwi-dong, Seogwipo-si, Jeju-do; locationRemarks: collected by SCUBA-diving; verbatimCoordinates: 33°13'31.06"N, 126°33'54.57"E; **Event:** eventDate: 26 Mar 2019
- **Type status:** Other material. **Occurrence:** individualCount: 1; occurrenceID: 8B88C27E-086E-5F4E-B663-D333BF8D776F; **Taxon:** scientificName: *Ischnochitonboninensis*; **Location:** country: Korea; locality: Is. Mun, Seogwi-dong, Seogwipo-si, Jeju-do; locationRemarks: collected by SCUBA-diving; verbatimCoordinates: 33°13'35.78"N, 126°34'07.27"E; **Event:** eventDate: 27 Mar 2019
- **Type status:** Other material. **Occurrence:** individualCount: 1; occurrenceID: 6A0AE6BA-B86B-5D25-A78E-A730101EC7B8; **Taxon:** scientificName: *Ischnochitonboninensis*; **Location:** country: Korea; locality: Geumgye-ri, Gogun-myeon, Jindo-gun, Jeollanam-do; verbatimCoordinates: 34°25'21.32"N, 126°20'56.94"E; **Event:** eventDate: 14 Dec 2016
- **Type status:** Other material. **Occurrence:** individualCount: 1; occurrenceID: 5DF8577C-F423-5E58-AF2D-9FB504D98202; **Taxon:** scientificName: *Ischnochitonboninensis*; **Location:** country: Korea; locality: Gujora Beach, Gujora-ri, Irun-myeon, Geoje-si, Gyeongsangnam-do; verbatimCoordinates: 34°48'05.58"N, 128°41'28.40"E; **Event:** eventDate: 12 Jun 2018
- **Type status:** Other material. **Occurrence:** individualCount: 1; occurrenceID: 1DB2075D-449C-5995-BCB5-B9A6A2EF4C92; **Taxon:** scientificName: *Ischnochitonboninensis*; **Location:** country: Korea; locality: Haengnam Lighthouse, Dodong-ri, Ulleung-eup, Ulleung-gun, Gyeongsangbuk-do; locationRemarks: collected by SCUBA-diving; verbatimCoordinates: 37°29'13.48"N, 130°55'00.18"E; **Event:** eventDate: 30 Oct 2018

#### Description

Body elongate-oval shaped, medium, less than 30 mm in length (Fig. 2A and C–E; in examined materials, body length [BL] 14.6–21.76mm, body width [BW] 7.21–12.23 mm). Valves generally yellowish-green to dark green or wholly whitish or pinkish in colour, brown streaks or cream, yellow, bright pink blotches. Girdle rather narrower in width than valves, dark brown, often with yellow-brown blotches or transverse bands. Gills arrangement holobranchial and adanal on both sides with interspace (Fig. 2B).

Valves: Head valve semicircular in shape, tegmentum sculpted with about 50 fine radial riblets and a few strong growth lines; radial riblets bifurcate towards margin, but faint towards apex; anterior margin round; posterior margin widely V-shaped (Fig. 3A). Intermediate valves broadly rectangular in shape, not beaked (Fig. 3B and C), dorso-ventrally rounded or subcarinated, somewhat high in frontal view, side slope slightly convex (Fig. 3J; elevation ratio of 0.46 in the 4^th^ valve); anterior margin nearly straight to slightly convex, except for 2^nd^ valve (Fig. 3B ; more convex than other intermediate valves, almost triangular); lateral margins somewhat round; posterior margin straight; tegmentum in central area (ca) with rows of densely small, elongate granules in quincunx or zig-zag patterns (Fig. 3I); lateral area (la) slightly raised, sculpted with 5–7 riblets, dividing to almost double towards margin, similar to head valve (Fig. 3B and C). Tail valve almost semicircular, as wide as head valve; anterior margin nearly straight (Fig. 3D); mucro (m) subcentral, not pointed; sculpture of antemucronal area (ama) similar to central area of intermediate valves; postmucronal area (pma) sculpted like head valve; postmucronal slope (pms) weakly concave (Fig. 3K). Articulamentum light blue, generally red violet in centre, very thin, reflecting colouration and patterns on tegmentum. Apophyses (ap; sutural laminae) short, delicate, triangular in 2^nd^ valve, but broad rectangular in the other valves; jugal lamina (jl) extremely short; jugal sinus (js) shallow, nearly straight; insertion plate (ip) short, teeth (t) relatively smooth with varying size; slit formula 10–13/1/9–10, slit rays (slr) distinct (Fig. 3E–H). Eaves (e) narrow, solid (Fig. 3J).

**Figure 7.**
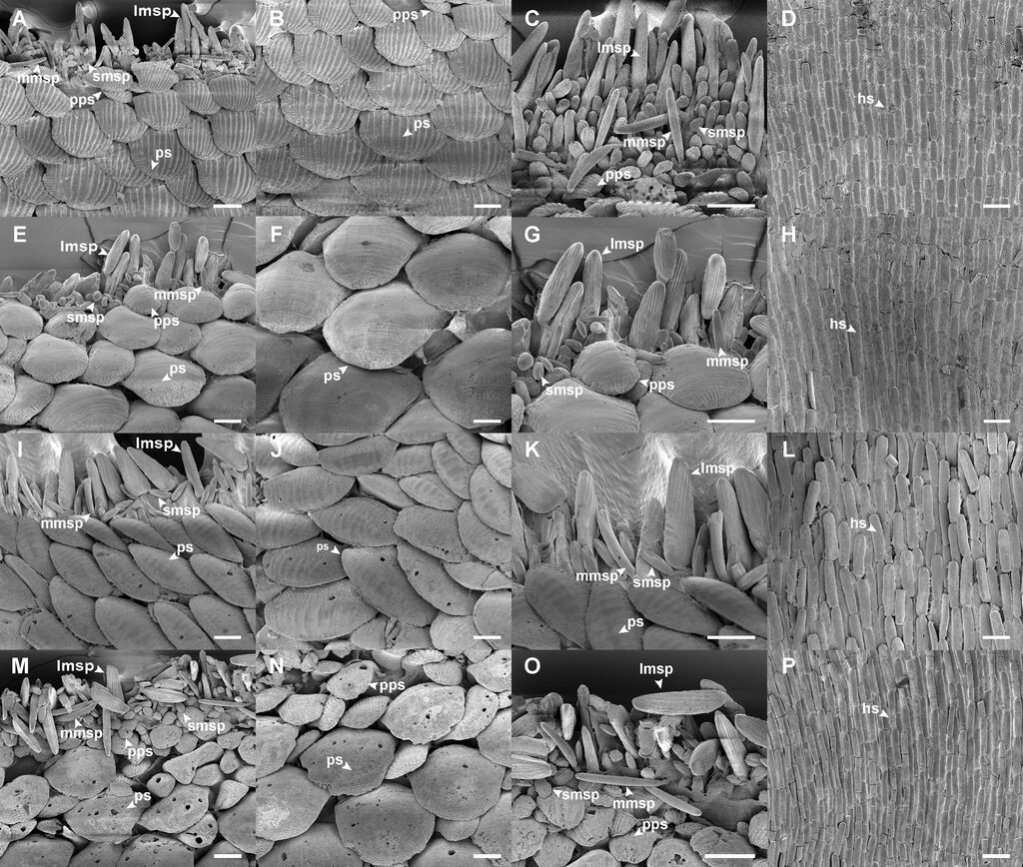
Microstructure of girdles in *Ischnochiton* species using scanning electron microscope. **A–D** *I.boninensis*; **E–H** *I.comptus* (NIBRIV0000863098); **I–L** *I.hakodadensis* (NIBRIV0000863046); **M–P** *I.hayamii* (NIBRIV0000863103); **A** perinotum scales and marginal spicules of *I.boninensis*; **B** perinotum scales of *I.boninensis*; **C** marginal spicules of *I.boninensis*; **D** hyponotum scales of *I.boninensis*; **E** perinotum scales and marginal spicules of *I.comptus*; **F** perinotum scales of *I.comptus*; **G** marginal spicules of *I.comptus*; **H** hyponotum scales of *I.comptus*; **I** perinotum scales and marginal spicules of *I.hakodadensis*; **J** perinotum scales of *I.hakodadensis*; **K** marginal spicules of *I.hakodadensis*; **L** hyponotum scales of *I.hakodadensis*; **M** perinotum scales and marginal spicules of *I.hayamii*; **N** perinotum scales of *I.hayamii*; **O** marginal spicules of *I.hayamii*; **P** hyponotum scales of *I.hayamii*. Abbreviations: hs, hyponotum scale; lmsp, large marginal spicule; mmsp, middle marginal spicule; pps, peripheral perinotum scale; ps, perinotum scale; smsp, small marginal spicule. Scale bars: **A–P** = 50 μm. The NIBR voucher specimen numbers are provided in parentheses.

Girdle: Perinotum scales (ps: length [L] 91–110.8 μm, width [W] 120.3–190.7 μm) small, oval, flat, slightly bent, sculpted with 9–16 fine longitudinal ribs converging to distal tip, densely overlapped, parallel to outer margin (Fig. 7A and B); peripheral scales of perinotum (pps: L 25.8–56.4 μm, W 33.8–86.5 μm) much smaller than perinotum scales, elongate-oval, with 4–7 fine, flat longitudinal ribs (Fig. 7A–C); marginal spicules of three types: large one (lmsp: L 67.2–87.6 μm, W 16.9 μm) flat-ovate, narrower to tip, blunt, with weakly longitudinal ribs, middle one (mmsp: L 68.2–80.3 μm, W 7.3–10 μm) long, nearly straight, smooth, rod-shaped with blunt tip and small one (smsp: L 22.1–33.5 μm, W 9.9–11.5 μm) very short, stout with strong radial ribs at tip (Fig. 7A and C); hyponotum scales (hs: L 56.7 μm, W 14.7 μm) hyaline, smooth, oblong, overlapped, radiated to outline (Fig. 7D).

**Figure 8.**
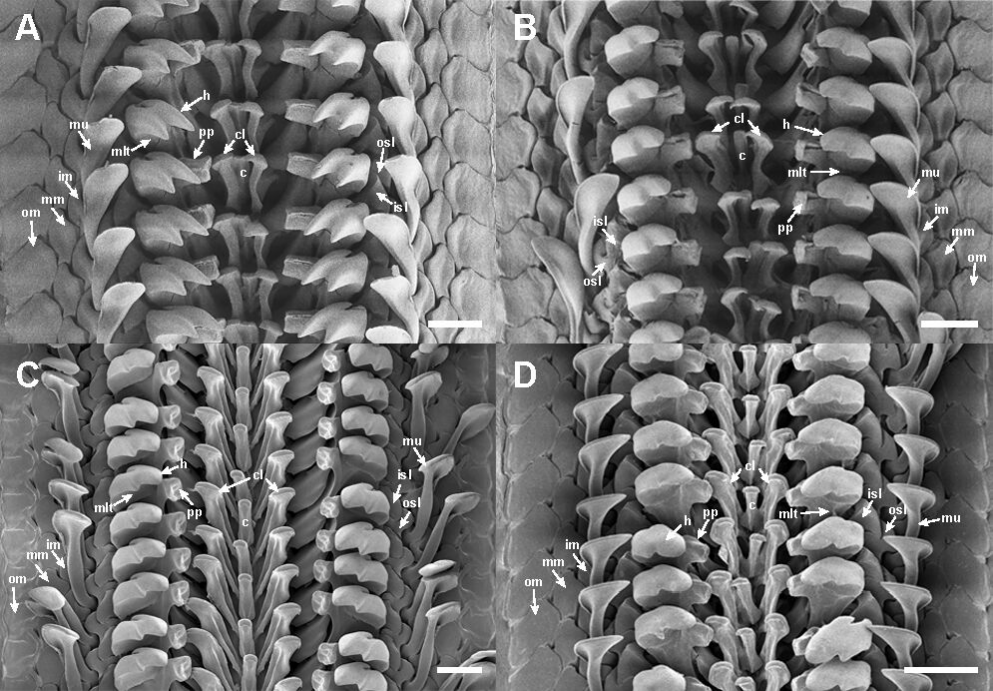
Microstructure of radula of *Ischnochiton* species using scanning electron microscope. **A** *I.boninensis* (NIBRIV0000863095); **B** *I.comptus* (NIBRIV0000863098); **C** *I.hakodadensis*; **D** *I.hayamii* (NIBRIV0000863103). Abbreviations: c, central tooth; cl, centro-lateral tooth; h, head of major lateral tooth; im, inner marginal tooth; isl, inner small lateral tooth; mlt, major lateral tooth; mm, middle marginal tooth; mu, major uncinus tooth, om; outer marginal tooth; osl, outer small lateral tooth; pp, petaloid process. Scale bars: **A–D** = 100 μm. The NIBR voucher specimen numbers are provided in parentheses.

Radula: Radula teeth symmetrical (Fig. 8A). Central tooth (c) small, narrow, keeled from basal, blade broader than stalk, deeply concaved, bent forward; centro-lateral teeth (cl) slightly taller than central tooth, cuspids thickened, convex; head (h) of major lateral teeth (mlt) bicuspid, cuspids sharp, tapering towards tip, inner cuspid longer than outer one; petaloid process (pp; wing) below at head of major lateral tooth, large, rectangular, inner distal concave; major uncinus teeth (mu) long, spatula-shaped with rounded tip; marginal teeth (im, mm, om) flat, increasing in size from inner to outer teeth.

#### Distribution

China, Hong Kong, Japan, Vietnam and Korea.

##### Type locality

Japan: Sagami Misaki and Bonin Islands (Ogasawara).

#### Taxon discussion

This species is morphologically similar to *I.comptus* (Gould, 1859), but easily distinguished by the size and microstructure of its perinotum scales (Table 2). *I.boninensis* has small perinotum scales sculpted with conspicuous fine longitudinal ribs (Fig. 7A and B), while *I.comptus* displays relatively large, smooth, glossy scales (Fig. 7E and F). In contrast to the markedly high individual variations in tegmentum colour and pigmentation patterns, the mtDNA *cox1* sequences of this species showed moderate within-species variations, less than 1.8% (Fig. 2A and C–E, Table 3), even amongst individuals sampled from different geographic regions (i.e. Japan and Korea).

### Ischnochiton comptus

(Gould, 1859)

0D36C7F6-B5DC-5D32-A63C-A5CD4377E52D

https://www.marinespecies.org/aphia.php?p=taxdetails&id=848047

Chiton (Leptochiton) comptus Gould (1859): 163–164; Gould (1862): 117 (partim).
Ischnochiton comptus : Pilsbry (1892): 117 (partim); 1895: 114; Thiele (1909): 111, 114; Taki (1938): 366–371, pl. 14, figs. 5, 10, pl. 15, figs. 6, 7, pl. 25, figs. 9–16, pl. 27, figs. 6, 7, pl. 29, figs. 11–16 (partim, =?boninensis); Lee (1956): 11; Kang (1971): 52; Yoo (1976): 146, pl. 35, fig. 9; Kaas and Van Belle (1980): 29; Kaas and Van Belle (1998): 49; Kira (1982): 183, pl. 67, fig. 1; Saito (1995): 105; Saito (2000): 12, 13, pl. 6, fig. 14; Saito (2017): 732, pl. 5, fig. 1; Slieker (2000): 98, 99, pl. 37, fig. 11; Owada (2016): 6, figs. 4F–J, 5E–H; Sirenko and Zhang (2019): 6, 7, fig. 2C.
Ischnochiton thaanumi Dall (1926): 66 (partim).
Ischnochiton (Ischnochiton) comptus : Taki (1962): 43; Higo (1973): 6; Yum (1988): 18–20, pl. 1, fig. 3, pl. 7, pl. 22, fig. 5, 6, pl. 28, fig. 3.
Ischnochiton (Ischnochiton) comptus (Gould) forma comptus : Taki (1964a): 347, text-figs. 1, 3, 5; 1964b: 408; Okada et al. (1981): 8; Higo and Goto (1993): 3.
Ischnochiton (Haploplax) comptus : Kaas and Van Belle (1994): 70–72, fig. 28, map. 7; Higo et al. (1999): 27; Lee and Min (2002): 94; Min et al. (2004): 71–73, fig. 9.

#### Materials

- **Type status:** Other material. **Occurrence:** individualCount: 4; occurrenceID: C27ECD33-0416-5309-AEAF-F17C6710DA7D; **Taxon:** scientificName: *Ischnochitoncomptus*; **Location:** country: Korea; locality: Gamsan-ri, Andeok-myeon, Seogwipo-si, Jeju-do; verbatimCoordinates: 33°14'02.18"N, 126°21'45.60"E; **Event:** eventDate: 26 Jan 2015
- **Type status:** Other material. **Occurrence:** individualCount: 3; occurrenceID: 1B2B3AA3-4F11-5273-AA4E-08B84F18B4E2; **Taxon:** scientificName: *Ischnochitoncomptus*; **Location:** country: Korea; locality: Gamsan-ri, Andeok-myeon, Seogwipo-si, Jeju-do; verbatimCoordinates: 33°14'06.31"N, 126°21'34.21"E; **Event:** eventDate: 22 Dec 2015
- **Type status:** Other material. **Occurrence:** individualCount: 2; occurrenceID: CDA06FBD-6774-5AB8-867C-BA22C65B8F5E; **Taxon:** scientificName: *Ischnochitoncomptus*; **Location:** country: Korea; locality: Dokgot-ri, Daesan-eup, Seosan-si, Chungcheongnam-do; verbatimCoordinates: 36°59'05.79"N, 126°19'50.10"E; **Event:** eventDate: 22 Mar 2016
- **Type status:** Other material. **Occurrence:** individualCount: 1; occurrenceID: 50DCB9E7-A1D5-5F61-B1D1-5AF5C4A05326; **Taxon:** scientificName: *Ischnochitoncomptus*; **Location:** country: Korea; locality: Jukbyeon-ri, Jukbyeon-myeon, Uljin-gun, Gyeongsangbuk-do; locationRemarks: collected by snorkeling; verbatimCoordinates: 37°03'33.85"N, 129°25'42.04"E; **Event:** eventDate: 08 Sep 2016
- **Type status:** Other material. **Occurrence:** individualCount: 2; occurrenceID: 1A911F9E-3306-522B-AF9A-26E6BF151A75; **Taxon:** scientificName: *Ischnochitoncomptus*; **Location:** country: Korea; locality: Sagye-ri, Andeok-myeon, Seogwipo-si, Jeju-do; verbatimCoordinates: 33°13'37.13"N, 126°18'30.92"E; **Event:** eventDate: 23 Apr 2017
- **Type status:** Other material. **Occurrence:** individualCount: 1; occurrenceID: 1D4D279E-F639-5747-9CE0-B5B98C3A7CCB; **Taxon:** scientificName: *Ischnochitoncomptus*; **Location:** country: Korea; locality: Hangaechang, Is. Mun, Seogwi-dong, Seogwipo-si, Jeju-do; locationRemarks: collected by SCUBA-diving; verbatimCoordinates: 33°13'31.06"N, 126°33'54.57"E; **Event:** eventDate: 26 Mar 2019

#### Description

Body oval-shaped, less than 25 mm in length (Fig. 2F and H–J; in examined materials, BL 15.69–19.38mm, BW 9.9–11.68mm). Valves overall light green to greyish-green in colour, partially white, cream, pink blotches or dots of various size. Girdle rather narrow, cream in colour with dark brown transverse bands. Gills arrangement holobranchial and adanal on both sides with interspace (Fig. 2G).

Valves: Head valve semicircular, tegmentum sculpted with weak microgranules; ca. 50 radial riblets gradually fainter towards apex; growth lines rather distinct; anterior margin round; posterior margin widely V-shaped (Fig. 4A). Intermediate valves broadly rectangular in shape, hardly beaked (Fig. 4B and C), dorso-ventrally subcarinated, moderately elevated in frontal view, side slope slightly convex (Fig. 4J; elevation ratio of 0.43 in 4^th^ valve); anterior margin nearly straight to slightly convex, except for 2^nd^ valve (Fig. 4B; triangular with rounded angle); lateral margins rounded; posterior margin straight; tegmentum in central area (ca) with low oval granules, arranged in quincunx or zig-zag patterns, converging and weaker towards apex (Fig. 4I); lateral area (la) somewhat raised, sculpted with 4–5 faint radial riblets (Fig. 4B and C). Tail valve almost semicircular, as wide as head valve; anterior margin convex (Fig. 4D); mucro (m) subcentral, not projecting; sculpture of antemucronal area (ama) like central area of intermediate valves; postmucronal area (pma) sculpted similarly to head valve, with more pronounced radial ribs; postmucronal slope (pms) straight (Fig. 4K). Articulamentum light greenish-blue, generally red violet in centre. Apophyses (ap; sutural laminae) short, rounded-angled subtrapezoid in shape, except for that of 2^nd^ valve, which is triangular; jugal lamina (jl) vert short; jugal sinus (js) shallow, about one-fourth width of valves, almost straight; insertion plate (ip) short, teeth (t) relatively smooth; slit formula 10/1/10, slit rays (slr) faint (Fig. 4E–H). Eaves (e) narrow, solid (Fig. 4J).

Girdle: Perinotum scales (ps: L 114.8–192.5 μm, W 146.7–232.7 μm) large, oval, slightly curved, imbricating, with faint concentric lines and indistinct scratches towards apex, parallel to outer margin (Fig. 7E and F); peripheral scales of perinotum (pps: L 59.6–73.7 μm, W 64.7–97.2 μm) smaller and narrower than perinotum scales (Fig. 7E, G); marginal spicules with three types: large one (lmsp: L 93.6 μm, W 20.7 μm) flat-ovate, narrowing towards tip, with fine feather-like grooves, middle one (mmsp: L 50.1 μm, W 11 μm) long, smooth, slightly curved rod-shaped and small one (smsp: L 17.8–26.4 μm, W 6.7–11.1 μm) very short, obese spheroidal (Fig. 7E, G); hyponotum scales (hs: L 71.2 μm, W 18.5 μm) hyaline, smooth, narrow, oblong, overlapped (Fig. 7H).

Radula: Radula symmetrical rows (Fig. 8B). Central tooth (c) narrow, blade wide, bent forward; centro-lateral teeth (cl) wing-like shape, each blade inwardly convex; head (h) of major lateral teeth (mlt) bicuspid, cuspid short, rounded-angled, inner one longer than outer one; petaloid process (pp; wing) small rectangular; major uncinus teeth (mu) tall, curving inwards with broad spatula-like tip; marginal teeth (im, mm, om) flat, with outer one largest.

#### Distribution

Australia, China, Hong Kong, Indonesia, Japan, Philippines, Taiwan, Vietnam and Korea.

##### Type locality

Japan: Oushima (Amami Oshima, Kagoshima Prefecture).

#### Taxon discussion

This species is considered taxonomically challenging due to its extremely high morphological variation, as well as recently discovered cryptic species. Recent studies, based on a combination of morphological and molecular analyses, have uncovered two cryptic species (Owada 2016, Owada 2018): *I.manazuruensis* and *I.hayamii*. They show very similar morphology to *I.comptus*, but are distinguished by the microstructure of the central area in the intermediate valves and the size of the girdle scales (Owada 2016, Owada 2018). In *I.comptus* and *I.hayamii*, the central area of the intermediate valves is covered with granules arranged in quincunx or zig-zag patterns, whereas *I.manazuruensis* shows an almost smooth surface in this area. Furthermore, the perinotum scales of *I.comptus* are similar in size to *I.manazuruensis* (300–400 μm), but larger than *I.hayamii* (150–200 μm). This species shows a moderate within-species sequence divergence of the mtDNA *cox1*, with a maximum divergence of 1.28%, but relatively high sequence divergence from their congeneric species compared in this study, ranging from 11.31% (*I.poppei*) to 16.70% (*I.hakodadensis*) (Table 3).

### Ischnochiton hakodadensis

P. P. Carpenter, 1893

588E02C7-295D-54B3-8053-F74D8BB69957

https://www.marinespecies.org/aphia.php?p=taxdetails&id=848089

Ischnochiton (Ischnoradsia) hakodadensis Carpenter in Pilsbry (1893): 147, pl. 19, figs. 64–66; Pilsbry (1895): 114; Taki (1962): 44; Taki (1964b): 409; Kaas and Van Belle (1980): 57; Yum (1988): 21, 22, pl. 2, fig. 1, pl. 9, pl. 23, fig. 3, 4, pl. 28, fig. 5.
Ischnoradsia hakodadensis : Taki (1938): 373–375, pl. 15, fig. 8, pl. 26, figs. 1–5, pl. 27, figs. 1–5, pl. 28, figs. 19–20.
Ischnochiton (Ischnochiton) hakodadensis : Taki (1962): 43; Kaas and Van Belle (1990): 180–182, fig. 81, map. 31; Dell'Angelo et al. (1990): 34, 35; Higo et al. (1999): 26; Lee and Min (2002): 94; Min et al. (2004): 70, 71.
Ischnochiton (Ischnoradsia) hakodatensis [sic]: Higo (1973): 6; Okada et al. (1981): 8; Higo and Goto (1993): 4.
Ischnochiton hakodadensis : Thiele (1909): 111, 112, taf. 8, fig. 44; Jakovleva (1952): 96, 97, fig. 47, pl. 8, fig. 3; Saito (1995): 103; Saito (2000): 12, 13, pl. 6, fig. 17; Saito (2017): 732, pl. 5, fig. 4; Slieker (2000): 98, 99, pl. 37, fig. 12; Qi (2004): 5, pl. 002E; Sirenko (2013): 148; Owada (2018): 285–287, figs. 2G, 3, 7B.

#### Materials

- **Type status:** Other material. **Occurrence:** individualCount: 6; occurrenceID: D3A30D7C-BDDB-582F-8CA6-CC8D5BE4CF67; **Taxon:** scientificName: *Ischnochitonhakodadensis*; **Location:** country: Korea; locality: Dokgot-ri, Daesan-eup, Seosan-si, Chungcheongnam-do; verbatimCoordinates: 36°59'05.79"N, 126°19'50.10"E; **Event:** eventDate: 22 Mar 2016
- **Type status:** Other material. **Occurrence:** individualCount: 1; occurrenceID: 6CB3AB45-7BDB-55F7-9A0E-21E1F02E87C5; **Taxon:** scientificName: *Ischnochitonhakodadensis*; **Location:** country: Korea; locality: Buk-ri, Deokjeok-myeon, Ongjin-gun, Incheon-si; verbatimCoordinates: 37°15'53.09"N, 126°06'00.36"E; **Event:** eventDate: 18 Apr 2018
- **Type status:** Other material. **Occurrence:** individualCount: 3; occurrenceID: CBF6FEED-0921-54F9-AA2B-F497D6CA6F3D; **Taxon:** scientificName: *Ischnochitonhakodadensis*; **Location:** country: Korea; locality: Ayajin-ri, Toseong-myeon, Goseong-gun, Gangwon-do; locationRemarks: collected by snorkeling; verbatimCoordinates: 38°16'13.35"N, 128°33'28.75"E; **Event:** eventDate: 14 May 2018
- **Type status:** Other material. **Occurrence:** individualCount: 4; occurrenceID: 3E051C4B-8B3C-55FC-B092-5897991E6801; **Taxon:** scientificName: *Ischnochitonhakodadensis*; **Location:** country: Korea; locality: Ayajin-ri, Toseong-myeon, Goseong-gun, Gangwon-do; locationRemarks: collected by snorkeling; verbatimCoordinates: 38°16'13.35"N, 128°33'28.75"E; **Event:** eventDate: 22 May 2019

#### Description

Body shape oval, rarely over 30 mm in length (Fig. 2K and M; in examined materials, BL 18.63–25.01mm, BW 10.92–15.66mm). Valves mostly dark green or greyish-brown, even reddish-brown in colour, with cream, yellow, blackish-brown stripes or blotches of various size. Girdle rather narrow, with alternating light and dark transverse bands. Gills arrangement holobranchial and adanal in both sides with interspace (Fig. 2L).

Valves: Head valve semicircular, tegmentum sculpted with ca. 65 fine radial riblets, cut into several distinct concentric growth lines; anterior margin round; posterior margin widely V-shaped; (Fig. 5A). Intermediate valves broadly rectangular in shape, not beaked (Fig. 5B and C), dorso-ventrally subcarinated, moderately elevated in frontal view, side slope convex (Fig. 5J; elevation ration of 0.39 in 4^th^ valve); anterior margin straight, except for 2^nd^ valve (Fig. 5B; convex, nearly triangular); lateral margins almost rounded; posterior margin straight; tegmentum in central area (ca) almost smooth with minute, low granules arranged in quincunx or zig-zag patterns (Fig. 5I); jugal area (ja) somewhat raised, separated from pleural area (pa) by slightly different elevation; lateral area (la) slightly elevated, sculpted with 6–8 fine radial riblets; concentric growth lines several, distinct across entire tegmentum (Fig. 5B and C). Tail valve almost semicircular, slightly narrower than head valve; anterior margin nearly straight in centre, convex on the both side (Fig. 5D); mucro (m) antemedian, not protruding; sculpture of antemucronal area (ama) like central area of intermediate valves; postmucronal area (pma) sculpted similarly to head valve; postmucronal slope (pms) steep, straight (Fig. 5K). Articulamentum light blue. Apophyses (ap; sutural laminae) short, rounded triangle or subtrapezoid in shape; jugal lamina (jl) very short; jugal sinus (js) shallow, about one-fourth width of valves, straight; insertion plate (ip) short, teeth (t) uneven in size, slightly roughened; slit formula 15/2–3/17, slit rays (slr) clearly marked (Fig. 5E–H). Eaves (e) very narrow, solid (Fig. 5J).

Girdle: Perinotum scales (ps: L 64–122 μm, W 124.9–203.5 μm) small, slightly convex, imbricating, nearly smooth or with faint longitudinal ribs, arranged diagonally to outer margin (Fig. 7I–K); marginal spicules with three types: large one (lmsp: L 100.5–116.5 μm, W 32.9–40.3 μm) flat-triangular, blunt tip, with fine feather-like grooves, middle one (mmsp: L 63.4–77.4 μm, W 10.5–11.4 μm) slender, smooth, slightly curved rod-shaped and small one (smsp: L 37.2–49.6 μm, W 11.7–14.8 μm) very short, obese rod-shaped with radial grooves on tip (Fig. 7I, K); hyponotum scales (hs: L 80.7–89.5 μm, W 17–23.2 μm) hyaline, smooth, elongated, rounded-edged rectangular, overlapped, arranged radially (Fig. 7L).

Radula: Radula symmetrical rows (Fig. 8C). Central tooth (c) small, narrow, blade slightly bent forward; centro-lateral teeth (cl) taller than central tooth, widening towards tip; head (h) of major lateral teeth (mlt) bicuspid, cuspid short, blunted, outer one shorter, pointed than inner one; petaloid process (pp; wing) short, rectangular with inwardly concave tip; major uncinus teeth (mu) slender with round, spoon-like head; marginal teeth (im, mm, om) flat, widening towards outer.

#### Distribution

China, Japan, Russia (Vladivostok) and Korea.

##### Type locality

Japan: Hakodadi (Hakodate, Hokkaido).

#### Taxon discussion

This species is relatively large in body size (> 30 mm) and easily distinguished from other congeneric species by the following characteristics. *I.hakodadensis* has 2–3 slits in the intermediate valves (Fig. 5F and G), whereas other *Ischnochiton* species have only one slit (Fig. 3F, G, Fig. 4F, G, Fig. 6F and G; Owada (2018)). Additionally, the perinotum scales are arranged diagonally to the outer margin in *I.hakodadensis* (Fig. 7I), while they are arranged parallel in other *Ischnochiton* species (Fig. 7A, E and M). It is noteworthy that these two morphological features distinguishing *I.hakodadensis* from other congeneric species are also present in many other *Lepidozona* species as well. Moreover, previous molecular phylogenetic analysis using four gene regions (COI, 16S, 18S and 28S) revealed that *I.hakodadensis* was not monophyletic, with some other *Ischnochiton* species grouped in a separate branch (Owada 2018). In contrast to the very low individual sequence variation (a maximum of 0.90%), this species shows very high genetic divergence from other congeneric species, ranging from 15.80% to 19.93% (Table 3). These findings, based on both morphological and molecular evidence, highlight the need for further studies to clarify the taxonomic position of this species within the family Ischnochitonidae. Furthermore, Owada (2018) described the accessory process of major lateral teeth in the radula, but these characters were not found in Korean *I.hakodadensis* specimens (Fig. 8C), requiring further study to confirm this discrepancy.

### Ischnochiton hayamii

Owada, 2018

A7930542-E652-5677-B047-581DACCC2C1C

https://www.marinespecies.org/aphia.php?p=taxdetails&id=1259313

Ischnochiton hayamii Owada (2018): 287, figs 2A–F, 4, 7A.

#### Materials

- **Type status:** Other material. **Occurrence:** individualCount: 2; occurrenceID: 5C753217-14FB-5D05-9BCF-8E0933B7989D; **Taxon:** scientificName: *Ischnochitonhayamii*; **Location:** country: Korea; locality: Sinam-ri, Seosaeng-myeon, Ulju-gun, Ulsan-si; verbatimCoordinates: 35°20'16.35"N, 129°19'10.12"E; **Event:** eventDate: 24 Nov 2016

#### Description

Body oval-shaped, small to medium in size (Fig. 2N; in examined material, BL 15.28 mm, BW 9.04 mm). Valves dark olive-green in colour, with small spots or large blotches of variable colours; cream, yellow, pink and brown. Girdle rather narrow, alternating with transverse bands of yellowish-brown and light yellow. Gills arrangement adanal and holobranchial on both sides with interspace (Fig. 2O).

Valves: Head valve semicircular in shape, tegmentum almost smooth with faint growth line; anterior margin round; posterior margin widely V-shaped (Fig. 6A). Intermediate valves broadly rectangular in shape, hardly beaked (Fig. 6B and C). Intermediate valves dorso-ventrally carinated, moderately elevated in frontal view and side slope nearly straight (Fig. 6J; elevation ratio of 0.4 in 4^th^ valve); anterior margin slightly convex, except for 2^nd^ valve (Fig. 6B; more convex than other intermediate valves, even appearing triangular); lateral margins somewhat round; posterior margin almost straight; tegmentum of central area (ca) densely arranged minute granules in quincunx or zig-zag patterns (Fig. 6I); lateral area (la) hardly raised, almost smooth like head valve (Fig. 6B and C). Tail valve semicircular, as wide as head valve; anterior margin straight (Fig. 6D); mucro (m) subcentral, slightly elevated; sculpture of antemucronal area (ama) same as central area of intermediate valves; postmucronal area (pma) nearly smooth such as head valve; postmucronal slope (pms) concave (Fig. 6K). Articulamentum light blue, central part of articulamentum red violet in colour. Apophyses (ap; sutural laminae) rectangular in shape, short, fragile and separated with very short jugal lamina (jl); jugal sinus (js) shallow, about one-third of tegmentum width; insertion plate (ip) teeth (t) uneven in size; slit formula 10/1/10, slit rays (slr) fine, but relatively distinct (Fig. 6E–H). Eaves (e) very narrow, solid (Fig. 6J).

Girdle: Perinotum covered with flat, oval, slightly bending, nearly smooth and overlapping scales (Fig. 7M, N; ps: L 108.2–157.6 μm, W 152.6–184.8 μm), scales arranged parallel to margin; peripheral scales of perinotum (pps: L 18.8–59.8 μm, W 79.5–92.9 μm) much smaller than perinotum scales, with 8–15 fine, flat longitudinal ribs (Fig. 7M–O); marginal spicules with three types: large one (lmsp: L 77.7–95.6 μm, W 23–27.34 μm) long, flat-ovate, narrower to tip, obtuse, with 5–7 weak longitudinal ribs; middle one (mmsp: L 97.7–100.9 μm, W 8.8–11.7 μm) long, slender, smooth, blunt rod-shaped; small one (smsp: L 18.3–21.6 μm, W 14.7–15.3 μm) very short, obtuse with strong radial ribs (Fig. 7M and O); hyponotum covered with hyaline, smooth, oblong and overlapping scales (Fig. 7P; hs: L 70.6–75.6 μm, W 11.3–17.5 μm).

Radula: Radula symmetrical rows with 17 teeth per transverse row (Fig. 8D). Central tooth (c) narrow oblong shape with forward bent blade; each centro-lateral tooth (cl) taller than central tooth, with wide inwardly convex cuspid; head (h) of major lateral teeth (mlt) bicuspid, inner cuspid rounded, outer one shorter and more angled than inner one; petaloid process (pp; wing) near base of major lateral tooth, blunt rod-shape, protruding slightly more than cuspids of major lateral tooth directed towards the centre; major uncinus teeth (mu) tall, slender, curving inwards with round spoon-like tip; marginal teeth (im, mm, om) flat, increasing size from inner to outer teeth.

#### Distribution

Japan (Zushi, Shimoda, and Hakodate) and Korea (Ulsan).

##### Type locality

Japan: Zushi, Kanagawa Prefecture.

#### Taxon discussion

Previously, three *Ischnochiton* species have been reported in Korea (National list of species of Korea 2022): *Ischnochitonboninensis*, *Ischnochitoncomptus* and *Ischnochitonhakodadensis*. *I.boninensis* and *I.comptus* are very similar in overall morphology and typically co-occur in sympatric regions in intertidal and subtidal habitats (Taki 1938). Owada (2018) noted that *I.hayamii* shares morphological similarities with these species in their overall appearance, moderated elevation and sculptures of the central area in the intermediate valves. Nevertheless, *I.hayamii* is distinguished from the other three Korean *Ischnochiton* species by their morphological differences in body size, back shape and sculptures of the lateral areas (see Table 2 for detailed comparison). Amongst the four Korean *Ischnochiton* species, *I.hayamii* is the smallest in adult body size (< 15 mm in length), whereas the other three species typically have larger sizes (*I.boninensis* and *I.comptus* range from 15–30 mm and *I.hakodadensis* is the largest species > 30 mm) (Kaas and Van Belle 1990, Kaas and Van Belle 1994, Owada 2016, Owada 2018). The dorsal back shape of *I.hayamii* is carinated (Fig. 6J), while it is rounded in *I.boninensis* (Fig. 3J) and subcarinated in both *I.comptus* and *I.hakodadensis* (Fig. 4J and Fig. 5J) (Pilsbry 1893, Kaas and Van Belle 1990, Kaas and Van Belle 1994, Owada 2018). In microstructures of the valves, the lateral areas are almost smooth with faint radial ribs in *I.hayamii* (Fig. 6B and C), whereas the other three species possess 5–8 bifurcating radial ribs (Fig. 3B, C, Fig. 4B, C, Fig. 5B and C) (Taki 1938, Kaas and Van Belle 1990, Kaas and Van Belle 1994, Owada 2016, Owada 2018).

## Discussion

### Morphological comparison amongst Korean Ischnochiton species

As documented in many other molluscan taxa, the morphological similarities shared amongst these *Ischnochiton* species, coupled with a high degree of morphological variation, make it challenging to accurately distinguish them based solely on morphological characteristics. The present study provides a comprehensive morphological comparison of four Korean *Ischnochiton* species by examining detailed characteristics, such as body size, back shape and the microstructures of the valves, girdle and radula (see Table 2 for detailed information). Amongst the Korean *Ischnochiton* species, *I.hayamii* is the smallest, with adult body sizes measuring less than 15 mm, while *I.boninensis* and *I.comptus* range from 15 to 30 mm and *I.hakodadensis* typically exceeds 30 mm. The back shape of *I.boninensis* is rounded, contrasting with the subcarinated shape observed in *I.comptus* and *I.hakodadensis* and the distinctly carinated shape found in *I.hayamii*. *I.boninensis*, *I.comptus* and *I.hakodadensis* possess 5–8 bifurcating radial ribs on the lateral areas of the intermediate valves, whereas *I.hayamii* has smooth lateral areas. In addition, *I.boninensis*, *I.comptus* and *I.hayamii* each have only one slit on the intermediate valve (Fig. 3F, G, Fig. 4F, G, Fig. 6F and G), which is typical of other *Ischnochiton* species, whereas *I.hakodadensis* has two or three slits (Fig. 5F and G). In the microstructures of the girdle, perinotum scales are arranged parallel to the outer margin in *I.boninensis*, *I.comptus* and *I.hayamii* (Fig. 7A, E and M), whereas, in *I.hakodadensis*, they are arranged diagonally to the outer margin (Fig. 7I). The perinotum scales in *I.boninensis*, *I.hakodadensis* and *I.hayamii* range from 150 to 300 ㎛ in width (Fig. 7A, B, I, J, M and N), whereas *I.comptus* has relatively larger scales, ranging from 300 to 400 ㎛ in width (Fig. 7E and F) (Taki 1938, Kaas and Van Belle 1990, Kaas and Van Belle 1994, Owada 2016, Owada 2018). The sculpture pattern of the perinotum scale varies amongst the four *Ischnochiton* species: in *I.boninensis*, the perinotum scale is sculpted with 8–18 conspicuous fine longitudinal ribs (Fig. 7A and B), whereas, in *I.comptus*, it is smooth (Fig. 7E and F). In *I.hakodadensis*, the perinotum scale is nearly smooth or sculptured with faint longitudinal ribs (Fig. 7I and J), while *I.hayamii*, it is almost smooth with weak, fine longitudinal ribs (Fig. 7M and N) (Pilsbry 1893, Taki 1938, Kaas and Van Belle 1990, Kaas and Van Belle 1994, Owada 2016, Owada 2018). In the radula morphology of the four Korean *Ischnochiton* species, the major lateral teeth are bicuspid, but the shape of the cusp differs slightly according to the species: *I.boninensis* (sharpened cusps; Fig. 8A), *I.comptus* (short and angled cusps; Fig. 8B), *I.hakodadensis* (blunt cusps; Fig. 8C) and *I.hayamii* (rounded cusps; Fig. 8D). These observations are consistent with morphological descriptions in previous literature (Taki 1938, Kaas and Van Belle 1990, Kaas and Van Belle 1994, Owada 2016, Owada 2018). Morphological comparisons presented in this study will provide valuable taxonomic information for distinguishing *Ischnochiton* species.

### Molecular phylogenetic analysis

**Figure 9.**
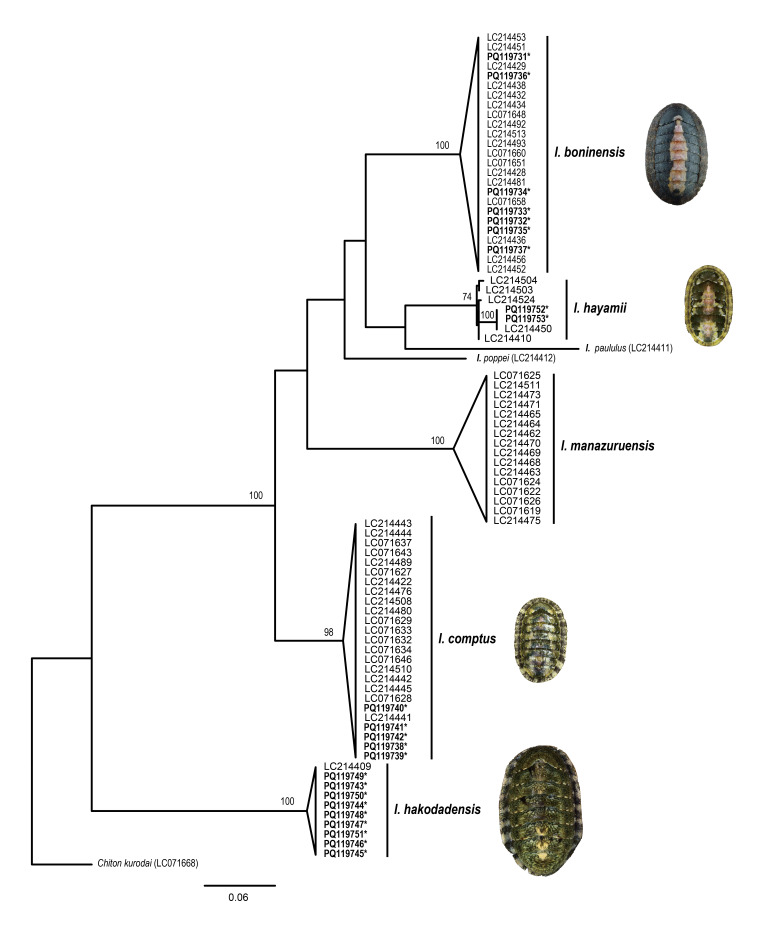
Phylogenetic relationships amongst north-western Pacific *Ischnochiton* species, based on the mtDNA *cox1* sequences, inferred from the Maximum Likelihood method. Node supporting values are indicated as bootstrap values (≥ 50). Sequences determined in this study are denoted by asterisks (*).

To verify species identification based on morphological data, molecular phylogenetic analyses were conducted using mtDNA *cox1* sequence data from four Korean *Ischnochiton* species (determined in this study) and additional sequences obtained from seven north-western Pacific (NWP) species documented in previous studies (Owada 2016, Owada 2018). The resulting phylogenetic tree shows that, irrespective of their geographic origins (i.e. Japan and Korea), all *cox1* sequences identified as the same species from morphological identification formed monophyletic groupings with strong bootstrap support values, each representing individual *Ischnochiton* species (Fig. 9). This result indicates that the mtDNA *cox1* sequences provide distinct resolution and serve as a useful genetic marker at the species level. However, the interrelationships amongst *Ischnochiton* species are unresolved, with most branches receiving low bootstrap support (≤ 50%). The lack of resolution in their phylogenetic relationships is likely due to the limited resolving power of the mtDNA *cox1* gene sequences in the phylogenetic study of *Ischnochiton* species. It is interesting to note that the NWP *Ischnochiton* species were not recovered as monophyletic within the family Ischnochitonidae in the previous analysis (Owada 2018) using a combined dataset of mitochondrial (*cox1* and 16S) and nuclear (18S and 28S rDNA) sequences. Further studies using independent molecular markers and extensive taxon sampling are necessary to accurately determine the interrelationships amongst NWP species within the phylogenetic framework of the family Ischnochitonidae.

## Supplementary Material

XML Treatment for Ischnochiton boninensis

XML Treatment for Ischnochiton comptus

XML Treatment for Ischnochiton hakodadensis

XML Treatment for Ischnochiton hayamii

## Figures and Tables

**Table 1.** Sampling localities and GenBank accession numbers of four Korean *Ischnochiton* species used for phylogenetic analyses in this study.

Species	Locality	Locality nos. ^(§)^	GenBank accession nos. (mtDNA *cox1*)
*I.boninensis*	Geumgye-ri, Gogun-myeon, Jindo-gun, Jeollanam-do	10	PQ119731
	Gujora-ri, Irun-myeon, Geoje-si, Gyeongsangnam-do	5	PQ119732
	Daejeong-eup, Seogwipo-si, Jeju-do	9	PQ119733
	Dodong-ri, Ulleung-gun, Gyeongsangbuk-do	2	PQ119734
	Jongdal-ri, Gujwa-eup, Jeju-si, Jeju-do	6	PQ119735
			PQ119736
			PQ119737
*I.comptus*	Dokgot-ri, Daesan-eup, Seosan-si, Chungcheongnam-do	11	PQ119738
			PQ119739
	Jukbyeon-ri, Jukbyeon-myeon, Uljin-gun, Gyeongsangbuk-do	3	PQ119740
	Andeok-myeon, Seogwipo-si, Jeju-do	8	PQ119741
			PQ119742
*I.hakodadensis*	Dokgot-ri, Daesan-eup, Seosan-si, Chungcheongnam-do	11	PQ119743
			PQ119744
			PQ119749
	Buk-ri, Deokjeok-myeon, Ongjin-gun, Incheon-si	12	PQ119745
	Ayajin-ri, Toseong-myeon, Goseong-gun, Gangwon-do	1	PQ119746
			PQ119747
			PQ119748
			PQ119750
			PQ119751
*I.hayamii*	Sinam-ri, Seosaeng-myeon, Ulju-gun, Ulsan-si	4	PQ119752
			PQ119753

^§^: The numbers correspond to the sampling locality numbers shown in Fig. 1.

**Table 2.** Morphological comparison among four Korean *Ischnochiton* species.

	*I.boninensis*	*I.comptus*	*I.hakodadensis*	*I.hayamii*
Body shape	elongate-oval	oval	oval	oval
Adult body size	medium (12.9–26 mm)	medium (15.7–19.4 mm)	medium (18.6–25 mm)	small (13–15.2 mm)
Dorsal elevation	moderate	moderate	moderate	moderate
Back shape (in frontal view)	rounded	subcarinated	subcarinated	carinated
Central area of intermediate valve	small, elongate granules in quincunx pattern	low, oval granules in quincunx pattern	almost smooth	minute granules in quincunx pattern
Lateral area of intermediate valve	slightly raised, with 5–7 radial ribs	somewhat raised, with 4–5 faint radial ribs	slightly raised, with 6–8 fine radial ribs and strong growth lines	hardly raised, almost smooth with faint radial ribs
Number of slit on intermediate valve	1	1	2–3	1
Mucro	subcentral	subcentral	antemedian	subcentral
Postmucronal slope	weakly concave	straight	steep, straight	concave
Perinotum scales	arranged parallel, small (150–250 μm), with 8–18 fine ribs	arranged parallel, large (300–400 μm), smooth	arranged diagonally, medium (200–300 μm), nearly smooth with faint ribs	arranged parallel, small (150–250 μm), almost smooth with weakly fine ribs
Radula: major lateral teeth	bicuspid, sharp	bicuspid, short, angled	bicuspid, blunted	bicuspid, rounded

**Table 3.** Uncorrected *p*-distances (%) for the mtDNA *cox1* sequences amongst seven north-western Pacific *Ischnochiton* species. The bold type indicates *p*-distances amongst individuals of the same species.

	*I.hayamii*	*I.boninensis*	*I.paululus*	*I.poppei*	*I.comptus*	*I.manazuruensis*	*I.hakodadensis*
*I.hayamii*	**0–2.15**						
*I.boninensis*	10.23–12.03	**0–1.80**					
*I.paululus*	11.85–13.11	13.82–14.72	–				
*I.poppei*	12.57–12.75	10.95–11.67	13.64	–			
*I.comptus*	12.21–14.18	12.03–13.11	13.64–14.36	11.31–12.03	**0–1.28**		
*I.manazuruensis*	12.75–15.08	12.03–13.82	15.08–15.80	14.00–15.44	11.49–13.46	**0.18–3.05**	
*I.hakodadensis*	15.80–16.70	15.98–16.88	19.57–19.93	15.80–16.16	15.80–16.70	16.34–17.41	**0–0.90**
